# Effects of Different Kinds of Physical Activity on Vascular Function

**DOI:** 10.3390/jcm13010152

**Published:** 2023-12-27

**Authors:** Francesca Saladini

**Affiliations:** Cardiology Unit, Cittadella Town Hospital, via Casa di Ricovero 40, 35013 Cittadella, Padova, Italy; saladinifrancesca@gmail.com

**Keywords:** exercise, aerobic physical activity, arterial stiffness, arterial distensibility, hypertension

## Abstract

Regular exercise is one of the main non-pharmacological measures suggested by several guidelines to prevent and treat the development of hypertension and cardiovascular disease through its impact on the vascular system. Routine aerobic training exerts its beneficial effects by means of several mechanisms: decreasing the heart rate and arterial pressure as well as reducing the activation of the sympathetic system and inflammation process without ignoring the important role that it plays in the metabolic profile. Through all these actions, physical training counteracts the arterial stiffening and aging that underlie the development of future cardiovascular events. While the role of aerobic training is undoubted, the effects of resistance training or combined-training exercise on arterial distensibility are still questioned. Moreover, whether different levels of physical activity have a different impact on normotensive and hypertensive subjects is still debated.

## 1. Introduction

Physical activity is universally recognized as one of the major non-pharmacological measures to reduce the risk of future cardiovascular events [1,2] and cardiovascular mortality [3]. The importance of physical activity was also confirmed in the latest guidelines released for the management and treatment of arterial hypertension [4], which recommended performing the equivalent of 10 metabolic task-hours per week of recreational physical activity, which corresponds to the suggested 150 min. per week of previous guidelines [5,6], in order to reduce the risk of future hypertension by 6%. The positive effects of exercise are not limited to a mere reduction in blood pressure (BP) values, but it also acts on the metabolic profile and weight reduction [7,8] and also at the microcirculation level, with an improvement in vascular function and structure [9]. However, it is still debated if the favorable effects of exercise are both for aerobic and resistance or combined training, if there are differences in acute and long-term effects and if the entity of the benefit is the same for hypertensive and normotensive patients.

## 2. Effects of Different Types of Exercise on Arterial Distensibility

**Figure 1:**
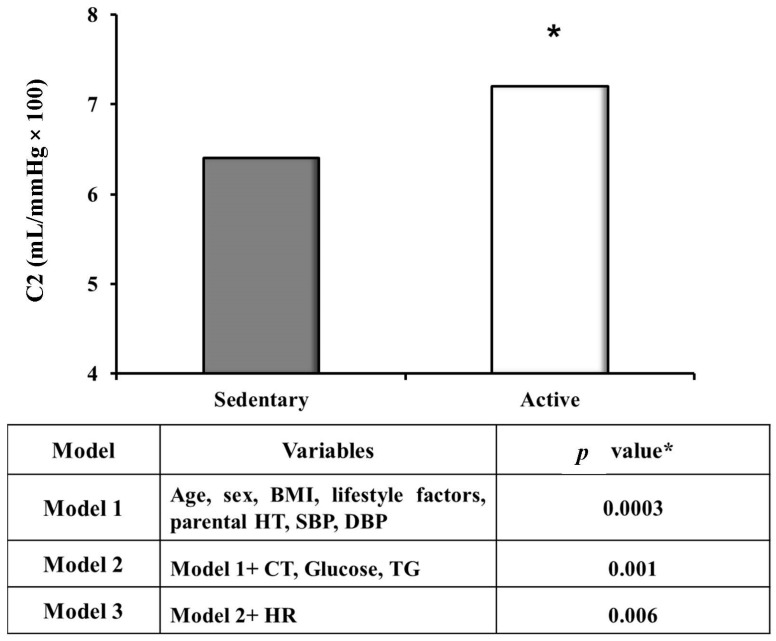
Baseline small-artery compliance in sedentary (n = 264) and active (n = 102) stage I hypertensive subjects from the HARVEST study. Adapted from Saladini F et al. [12]. BMI, body mass index; lifestyle factors: smoking habits, alcohol and coffee consumption and physical activity; parental HT, parental hypertension; SBP, systolic blood pressure; DBP, diastolic blood pressure; CT, total cholesterol; Tg serum triglycerides; HR, heart rate. *p* * value adjusted for age and sex.

Several published papers have highlighted the beneficial role of aerobic training on arterial hypertension [8,10,11] and endothelial function [12,13]. Palatini et al. [8] examined 796 young-to-middle-age untreated hypertensive subjects from the HARVEST study, divided into 153 exercisers and 493 sedentary subjects. As expected, the active subjects presented a lower 24 h BP (78.8 vs. 81.2 mmHg, *p* < 0.001 adjusted for body mass index (BMI), alcohol consumption and smoking habits), diurnal diastolic BP (80.4 vs. 83.3 mmHg, *p* < 0.0001 adjusted for BMI, alcohol consumption and smoking habits) and heart rate (24 h 68.2 ± 0.7 vs. 72.0 ± 0.4 bpm, *p* < 0.0001; diurnal 70.1 ± 0.7 vs. 74.5 ± 0.4 bpm, *p* < 0.0001; nocturnal 59.7 ± 0.7 vs. 62.0 ± 0.4 bpm, *p* = 0.004 adjusted for age, BMI, alcohol intake and smoking habits) in comparison to the sedentary ones. As mentioned above, the efficacy of aerobic exercise for these young patients was due to several components, such as weight loss (BMI among exercisers was 25.2 ± 0.3 vs. 26.2 ± 0.2 kg/m^2^, *p* = 0.002) and also the reduction in the activity of the sympathetic nerve system, as documented by the decrease in urinary norepinephrine at the 24 h urinary collection (norepinephrine/creatinine: 44.2 ± 2.6 μg/24 h vs. 8.9 ± 2.9 μg/24 h, respectively, *p* = 0.04, as also documented in the subsequent analysis) [14]. As mentioned above, aerobic training has a crucial role in the improvement in arterial function. Again, evidence from the HARVEST study [12] showed the positive impact of regular aerobic exercise during 6 years of follow-up among 366 young-to-middle-age subjects (n = 264 sedentary and n = 102 physically active), as examined through the use of radial applanation tonometry to detect large- and small-artery distensibility, peripheral resistances and the augmentation index. The authors observed that both at the enrolment (Figure 1) and at the final evaluation (Table 1), arterial distensibility parameters were higher for small- and large-artery compliance and lower for peripheral resistances and augmentation indexes among the physically active subjects compared to those who were not active in sports. However, these results only remained statistically significant at the final assessment for small-artery compliance after the inclusion of age and sex in the model [12], for the augmentation index when calculated with one-way repeated-measures ANOVA analysis (*p* for the interaction of active vs. sedentary × basal versus follow-up was 0.020) and for total peripheral resistance (*p* for interaction = 0.045) [12]. As documented for the positive effect of aerobic exercise on BP reduction, the mechanisms suggested to be underlying the positive effect of aerobic training on arterial distensibility were again the decreased heart rate of the active subjects (71.2 ± 8.9 bpm) compared to sedentary ones (76.6 ± 9.7 bpm; *p* < 0.001), which is a well-known, strong component of endothelial function according to previous published data [15,16,17], and the decrease in activity of the sympathetic nerve system [18], which may counteract the vasoconstriction phenomenon at peripheral sites and inhibit the chronic effect of sympathetic activation on the vascular tree. Also, Vriz et al. [13], in a recently published paper, confirmed the efficacy of aerobic exercise on endothelial function. The authors examined 120 leisure-time exercisers, 120 competitive athletes and 120 sedentary subjects who served as controls, who underwent an echo-tracking ultrasound system to assess carotid artery stiffness by means of the pressure–strain elastic modulus and one-point pulse wave velocity. The pressure–strain elastic modulus was reduced among those who were physically active (both groups) in comparison to those who were not active in sports (*p* < 0.03), as was the pulse wave velocity (*p* < 0.02). Moreover, in a multivariate regression analysis, physical activity was discovered to be a significant predictor of both the pressure–strain elastic modulus (*p* = 0.001) and pulse wave velocity (*p* < 0.001), even if both the associations were attenuated after the inclusion of heart rate in the model (*p* = 0.042 and 0.007, respectively). Again, this paper highlighted the important role of physical activity for heart rate as a mediator of the effects on vascular function [13], combined with receding microvascular remodeling, normalization of the capillary density, ameliorating the function of the vascular tree and, finally, counteracting the oxidative stress phenomenon [19,20,21,22]. All the evidence mentioned above refers to the efficacy of aerobic training, but what evidence do we have for different kinds of exercise? In the 2018 guidelines for arterial hypertension [5], isometric and resistance training were also recognized to be effective for the reduction in BP levels according to data reported in several randomized clinical trials [23,24,25], and it was also confirmed in the new guidelines [4]. For example, in the meta-analysis conducted by Cornelissen et al., a significant BP reduction of −3.9 (−6.4; −1.2)/−3.9 (−5.6; −2.2) mm Hg (*p* < 0.001) was clearly demonstrated, with a larger decrease among those who performed isometric handgrip training: −13.5 (−16.5; −10.5)/−6.1 (−8.3; −3.9) mmHg [23]. With regard to the effects of different exercise programs on vascular function, some evidence came from Ashor et al. [26]. In this meta-analysis involving 42 studies with an overall number of 1627 participants, the authors investigated the role that different types of physical training (aerobic, resistance or combined) had on vascular function, as detected by means of pulse wave velocity and the augmentation index. The authors observed that both parameters were significantly improved by aerobic training (−0.63 m/s, 95% CI: −0.90, −0.35, *p* < 0.01 for pulse wave velocity; −2.63%, 95% CI: −5.25 to −0.02, *p* = 0.05 for augmentation index), while they were unaffected by resistance training (−0.04 m/s, 95% CI: −0.42, to 0.34, *p* = 0.82 for pulse wave velocity; −1.69%, 95% CI: −4.11 to 0.72, *p* = 0.17 for augmentation index) or combined aerobic/resistance exercise programs (−0.35 m/s, 95% CI: −0.82, 0.12, *p* = 0.15, respectively). These results seem to be in contrast to what was previously observed by Miyachi et al. [27], who found an increase of 14.3% (95% CI 8.5% to 20.1%; 71%; heterogeneity, *p* < 0.001) in stiffness indexes (carotid arterial β stiffness and pulse wave velocity) in comparison with a group of controls, even if this increase seemed to be peculiar to young but not to middle-aged individuals, and of high-intensity resistance training, while resistance training did not affect changes in vascular function. On the opposite side, resistance training performed at a high-intensity level was significantly associated with an increase in stiffness of 11.6% [27]. Moreover, as shown in the meta-analysis conducted by Ashor et al. [26], some studies that investigated some peculiar types of resistance training were included, showing a favorable impact or at least no increase in arterial stiffness such as with lower intensity rather than high intensity training [28], as was also observed by Miyachi et al. [27] with lower-limb rather than upper-limb training [29], eccentric rather than concentric resistance training [30] and the combination of resistance with aerobic training [31]. However, despite the conflicting data regarding the role of combined aerobic and resistance training on vascular function, its proven beneficial effect on the cardio-metabolic system makes resistance training a fundamental adjunct to aerobic physical activity, especially for those individuals with metabolic syndrome [32,33].

## 3. Effect of Short-Term versus Long-Term Physical Activity on Arterial Distensibility

**Figure 2:**
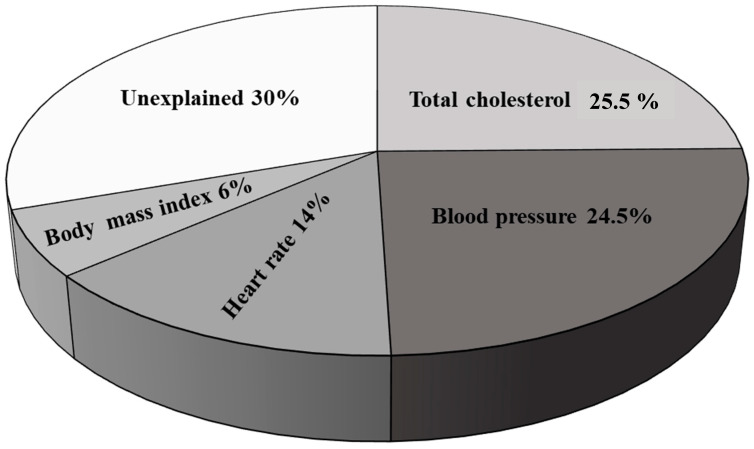
Contributing role that each single parameter exerts in mediating the relationship between thickening of the intima-media at the carotid level and aerobic training. Adapted from Palatini P et al. [39].

The positive long-term effect of physical activity on BP reduction and the prevention of target organ damage in hypertensive patients is not questionable. Vriz et al. [34] clearly demonstrated the efficacy of maintaining the same level of aerobic exercise for 3 months on BP levels. They compared 331 male non exercisers, 192 male mild exercisers and 49 male heavy exercisers and found an important reduction in diurnal systolic BP among the subjects who performed physical activity at a high-intensity level in comparison to inactive subjects (135.4 ± 0.6 mmHg among heavy exercisers, 134 ± 0.8 among mild exercisers, 132.2 ± 1.6 mmHg among sedentary subjects; *p* < 0.05 sedentary patients compared to heavy exercisers), demonstrating the beneficial effect of exercise training in reducing BP levels, which persisted through the three months of follow-up, during which the patients went on to perform the same level of exercise [34]. Similarly, in a review article, Cardoso et al. [35] concluded that both single episodes of aerobic training and chronic aerobic training were beneficial in terms of ambulatory BP reduction. In contrast, the beneficial effect of resistance training was demonstrated in the short term but not in the long term [35]. Moreover, long-term physical activity also showed a beneficial effect on hypertension mediated target organ damage [36,37,38,39,40]. Again, data from the HARVEST study [36] demonstrated among 454 untreated stage I hypertensives that regular aerobic physical activity over 8.3 years was associated with a lower left-ventricle mass among exercisers (39.0 ± 7.2 g/m^2.7^) in comparison to sedentary subjects (41.4 ± 9.0 g/m^2.7^; *p* = 0.02). Moreover, an increase in left-ventricular mass among sedentary subjects was detected during the follow-up (1.4 ± 6.5 g/m^2.7^), while no significant progression was observed among the active ones (0.6 ± 7.1 g/m^2.7^; *p* = 0.03). In a multivariate regression analysis, adjusted for several clinical confounders including age, sex, BMI, systolic and diastolic BP, hypertension duration, parental hypertension, follow-up length, smoking habits, coffee and alcohol intake, baseline left-ventricle mass, BP and weight changes during follow-up, aerobic physical training was a negative predictor of the increment in left-ventricular mass (O.R 0.26, 95 %CI 0.07–0.09, *p* = 0.033) [36]. According to the literature, several mechanisms have been proposed for the favorable effect of exercise on hypertensive-mediated organ damage. As mentioned above, one of the main determinants is the BP reduction induced by regular physical activity [41,42] followed by the reduction in body weight [43,44], improvement in vascular function and cardiac structure [37] as well as favorable modifications to metabolic parameters such as the increment of insulin sensitivity, a reduction in the activity of the renin–angiotensin–aldosterone system [40,45] and the sympathetic nerve system [7,14]. The efficacy of regular aerobic exercises in the long term was also described at the carotid artery level. Palatini et al. [39] demonstrated that patients who performed regular physical training at the end of a follow-up that lasted 6.5 years showed a reduction in the mean intima-media thickness (*p* = 0.01) at every single site, including the common carotid artery (*p* = 0.01), bulb (however, not significant *p* = 0.13) and internal carotid artery (*p* = 0.05), as well as in the maximum intima media thickness (*p* = 0.006), again at every single site, including the common carotid artery (however, not significant *p* = 0.09), bulb (again, not reaching the level of statistical significance *p* = 0.21), internal carotid artery (*p* = 0.01), in comparison to sedentary ones. In addition, the authors investigated the role that each single parameter has in the beneficial correlation between exercise and slowing the process of thickening of the intima-media at the carotid artery (Figure 2) and found that the main contributor was the reduction in total cholesterol, arterial pressure, heart rate and BMI [39]. This evidence confirmed the important role of regular physical training in counteracting the arterial stiffening of large vessels, such as the carotid artery, in young-to-middle-aged subjects with stage I hypertension through the important and beneficial modifications that act on the main components of the enhanced risk for cardiovascular disease [39]. The efficacy of long-term of physical activity was also demonstrated at the level of small vessels, confirming the fundamental role of exercise in contrasting the arterial aging phenomenon. This was shown by Saladini F et al. [12], not only at enrolment (Figure 1) but also after 6 years of follow-up (Table 1). Physically active (n = 102) subjects presented better distensibility parameters compared to subjects that did not regularly perform physical training (n = 264). However, the differences at the end of follow-up only reached statistical significance for small-vessel compliance after adjusting for age and gender [12]. The authors also investigated the longitudinal changes in the augmentation index and total peripheral resistances and found a significant time x group interaction (*p* for active vs. sedentary x baseline versus follow-up was 0.020 and 0.045, respectively [12]). One of the main determinants of the favorable effect of physical training on vascular function is the lower heart rate in trained individuals. According to the literature, the role of heart rate has been controversial for a long time, as previous findings seemed to advocate a cross sectional relationship between heart rate and arterial stiffness. In fact, according to a previous analysis, it was observed that the gradual increment in the heartbeat obtained through the stimulus of a cardiac pacemaker led to a consensual and gradual increment in the velocity of the pressure arterial wave [15,46]. A positive association between heart rate and pulse wave velocity was also found in a longitudinal study, as document by Tomiyama et al. [47] and by Benetos et al. [48], both in patients with normal or increased BP levels. Some clarifications on this issue came from Palatini et al. [49], who investigated the role of heart rate in determining changes in vascular function in the brief term and after several years of follow-up. The authors confirmed the negative cross-sectional correlation (r = −0.407, *p* = 0.001) between office heart rate and augmentation index. However, in the long term, a positive association was found between the ambulatory heart rate and the augmentation index (*p* = 0.0006), central BP (*p* = 0.014) and the amplification of BP from central to peripheral sites (*p* = 0.004) [49], indicating that the decrease in heart rate in the long term had a favorable impact on arterial stiffening, giving an important demonstration as to how regular exercise, through the reduction in the heartbeat in the long term, plays an important role in the decrease in the risk of cardiovascular morbidity and mortality among hypertensive subjects [50,51,52]. Important mechanisms in microcirculation may also account for the beneficial long-term effects of physical activity on endothelial function. As shown by De Ciuceis et al. [9] in a recent review, physical activity had a crucial role in counteracting vascular remodeling at the level of microcirculation sites among patients with elevated BP levels through the reduction in inflammation and fibrosis and neo angiogenesis, thereby promoting vasodilation and the restoration of the normal activity of the adipose tissue. Due to all these favorable mechanisms, aerobic exercise decreases vascular resistances at peripheral sites and enhances hematic flow in the vessels, preventing the impairment of arterial function.

## 4. Effect of Regular Exercise on Vascular Function in Hypertensive and Normotensive Patients

Another debated issue is the potential different effects of regular physical activity in normotensive and hypertensive subjects and the role played by exercise intensity. This topic was explored by Vriz and coworkers [13] in a multifactorial analysis by comparing sedentary subjects with subjects performing leisure physical activity and vigorous exercise who were hypertensive and normotensive. The authors observed a different effect of physical activity according to BP status. A progressive decrease in the pressure–strain elastic modulus (*p* = 0.009) and pulse wave velocity (*p* = 0.003) was found across the different levels of physical activity (sedentariness, leisure activity, competitive sport activity). In contrast, within subjects with high BP levels, reduced values of both the pressure–strain elastic modulus and pulse wave velocity were observed among those who performed leisure-time physical activity, while no beneficial effect was found among those who performed vigorous activity. Moreover, in a two-way ANOVA, a statistically significant association was observed between physical activity level and BP status for the elastic modulus (*p* = 0.03), while the association with the pulse wave velocity did not reach the level of statistical significance (*p* = 0.06). The apparent lack of any effect of strenuous physical activity was explained by an increase in oxidative stress induced by vigorous physical activity in hypertensive subjects, which was in agreement with previous findings [53,54,55,56]. This study was in line with previous ones that showed a non-beneficial effect of higher aerobic fitness on the carotid-to-femoral pulse wave velocity among middle-age and older treated hypertensives [57] and no differences for the aortic wave velocity among fit or unfit hypertensive subjects [58].

## 5. Role of Physical Activity in the Elderly 

Another interesting and debated issue is the role of physical activity among elderly subjects. In particular, it is unclear whether regular training at this age may have beneficial effects on vascular function. In older subjects, physiological vascular stiffening due to aging is associated with several comorbidities, including cardiovascular disease, atrial fibrillation, aortic stenosis, ischemic stroke, chronic kidney disease, cognitive impairment, frailty [59,60] and sarcopenia [61]. Whether regular physical activity exerts a positive effect of counteracting the aging process of the vessel wall is poorly known. Some evidence was provided by Vizzi et al. [62], who examined 26 subjects aged from 66 to 92 years performing regular physical activity three times/week. After 8 months, the authors observed no changes in most participants. A mild improvement in their neurodegenerative diseases was found in only two subjects. Similarly, Park et al. [63] observed that a regular program of aquatic exercises, performed in elderly patients with peripheral artery diseases, was useful in reducing the pulse wave velocity and ameliorating exercise capacity, as documented by the improvement in the six-minutes walking test. A beneficial effect of exercise for contrasting vascular stiffening due to aging was also found on top of using pharmacological drugs [64]. Fung et al. [64] examined 478 subjects with a mean age 68.6 years who were non-demented and with a high vascular risk who underwent to two different strategies to preserve cognitive function: drug treatment and/or physical exercise that included mind–body training or strenuous training. Interestingly, both types of exercise, combined with medication (H.R. 2.9 (1.1–7.7) *p* = 0.029 for mind–body training, H.R. 2.4 (1.1–5.3) 0.036 for strenuous exercise), were superior to using medication alone (*p* = n.s.) in counteracting cognitive decline. 

The above findings have important clinical implications suggesting that regular exercise also has a beneficial effect in elderly subjects, as stated in the Guidelines for international exercise recommendations in older adults [65]. These guidelines detail the intensity, frequency and types of exercises suitable for older people, including aerobic exercise as well as balance activities, in order to prevent cardiovascular disease, improve frailty and counteracting cognitive decline [65]. 

## 6. Conclusions

Regular exercise training, as suggested by several studies [1,2,4,5,6], is one of the main non-pharmacological measures to prevent the development of major adverse cardiovascular events due to its favorable impact on heart rate, BP and several components of metabolic syndrome.

Several mechanisms account for these beneficial effects, including the reduction in the activities of the sympathetic nerve system and the renin–angiotensin–aldosterone system and the inflammation process, leading to an improvement in endothelial function and vascular elasticity. The favorable effect of resistance training on endothelial function and large arteries remains somewhat controversial. However, there is evidence showing that this kind of exercise has a positive effect on the metabolic profile (for example, on insulin sensitivity and glucose control [33]), as well as on some measures of endothelial function, such as flow-mediated dilation [66]. These effects make the combination of both aerobic and resistance training the best method for achieving optimal cardiovascular fitness [32]. A clear example of how the different modalities of exercise should be combined in order to obtain the best beneficial effect in terms of cardiovascular protection comes from the meta-analysis conducted by Zhang and co-workers [67]. The authors examined 38 articles involving an overall number of 2089 subjects with cardiovascular disease who underwent aerobic, resistance or combined physical exercise. Each type of exercise led to an improvement in arterial and cardiac functions; aerobic function significantly reduced the aortic systolic pressure and augmentation index, improved pulse wave velocity, cardiac output and the ejection fraction, while resistance training had a beneficial effect on the aortic systolic and diastolic pressure, and combined exercise had a favorable effect on the pulse wave velocity and cardiac output. The authors concluded that a tailored program of different kinds of exercise should be prescribed in order to obtain the maximum beneficial effect on the cardiovascular system.

The favorable effect of intense physical activity seems to be greater among normotensives, while the effect among hypertensives is beneficial only at a lower intensity level. An exercise program should be implemented in all healthy subjects, in particular in those at higher risk of developing hypertension and cardiovascular disease, in order to counteract all micro- and macrovascular complications that may progress to manifesting disease. But exercise, in particular combined exercise [32], should not be denied even to people with overt cardiovascular disease [68], provided it is performed to a light-to-moderate intensity level. However, the major challenge for health care personnel is still to persuade patients to continue with their program of regular training, as the compliance to exercise is very poor [69,70]. Yet, even small repetitions of exercise may counteract the detrimental effects of sedentary behavior [71,72]. In an interesting randomized cross-sectional trial, Fryer and coworkers demonstrated that the simple repetition of a small movement of the leg, such as flexing and extending the feet, after a meal rich in fat, may have an important positive impact on vascular stiffness. Among those subjects who continued to be sedentary for three hours, there was a significant increase in the pulse wave velocity and other indexes of local arterial stiffness, while among those who interrupted the sedentary behavior with 5 min of interval exercises, there was a significant decrease in the augmentation index [71]. Similarly, Horiuchi et al. demonstrated that the interruption of the sitting position by means of leg exercises such as squats [72,73] and or calf raises [73] at twenty-minute intervals had a beneficial impact on arterial stiffness, as mediated by a decrease in blood glucose levels [72].

In conclusion, regular physical exercise, independently of the kind of exercise, has a fundament role in counteracting vascular aging and arterial dysfunction and must be encouraged in every subject in order to prevent future hypertension and cardiovascular events.

## Figures and Tables

**Table 1:** Follow-up evaluation of vascular function and hemodynamic indexes in the active verus stage 1 hypertensive participants from the HARVEST study. Adapted from Saladini F et al. [12].

Variables	Sedentary Subjects (n = 110)	Active Subjects (n = 42)	*p* *
C1, mL/mmHg × 10	16.4 ± 4.5	17.0 ± 4.8	n.s.
C2, mL/mmHg × 100	6.2 ± 2.8	7.9 ± 2.5	0.009
AIx, %	25.8 ± 22.6	14.5 ± 0.24	n.s.
Carotid-radial PWV, m/s	8.8 ± 2.1	9.1 ± 2.2	n.s.
Peripheral resistance, dyne × s × cm^−5^	1466.0 ± 236	1357.0 ± 228	n.s.
Central SBP, mmHg	120.4 ± 15.0	118.2 ± 10.9	n.s.

Data are presented as mean ± standard deviation. C1, large-artery compliance; C2, small-artery compliance, AIx, augmentation index, PWV, pulse wave velocity; SBP, systolic blood pressure. *p* * value adjusted for age and sex; n.s., not statistically significant (*p* > 0.05).
